# 静脉滴注异麦芽糖酐铁治疗缺铁性贫血的疗效及安全性评估：单中心回顾性分析

**DOI:** 10.3760/cma.j.cn121090-20230718-00009

**Published:** 2024-02

**Authors:** 少雪 丁, 伊慧 赵, 婷 王, 晶 关, 莉民 邢, 鸿 刘, 国锦 王, 晓明 王, 玉红 吴, 文 瞿, 嘉 宋, 化泉 王, 丽娟 李, 宗鸿 邵, 蓉 付

**Affiliations:** 天津医科大学总医院血液内科，天津市骨髓衰竭及癌性造血克隆防治重点实验室，天津 300052 Department of Hematology, Tianjin Medical University General Hospital, Tianjin Key Laboratory of Bone Marrow Failure and Malignant Hemopoietic Clone Control, Tianjin 300052, China

**Keywords:** 缺铁性贫血, 静脉异麦芽糖酐铁, 静脉蔗糖铁, Iron deficiency anemia, Intravenous ferric derisomaltose, Intravenous iron sucrose

## Abstract

**目的:**

探讨缺铁性贫血（IDA）患者应用异麦芽糖酐铁注射液对比蔗糖铁注射液治疗的临床疗效及安全性。

**方法:**

选取2021年6月至2023年3月期间收治的缺铁性贫血患者120例，应用异麦芽糖酐铁注射液静脉补铁治疗，观察患者治疗前后HGB提升的有效性和安全性。同时与同期病房应用蔗糖铁补铁患者临床疗效及安全性进行对比。

**结果:**

两组患者基线HGB相当。治疗12周内，异麦芽糖酐铁组提升HGB水平高于蔗糖铁，各个时间差异均具有统计学意义（均*P*<0.01），治疗4周患者HGB提升超过20 g/L比例更高（98.7％对75.9％）。治疗过程中，异麦芽糖酐铁组发生轻度不良反应比例略低于蔗糖铁组，两组均无严重不良输液反应发生，输注治疗过程中患者耐受性良好，无注射部位疼痛及色素沉着等不良反应。

**结论:**

IDA患者应用异麦芽糖酐铁治疗疗效满意，具有见效迅速、疗效确切、安全性高的优点，值得临床广泛应用。

## Abstract (en)

**Objective:**

To investigate the clinical efficacy and safety of ferric derisomaltose injection versus iron sucrose injection in the treatment of iron deficiency anemia (IDA).

**Methods:**

A total of 120 patients with iron deficiency anemia admitted from June 2021 to March 2023 were given intravenous iron supplementation with ferric derisomaltose to assess the efficacy and safety of hemoglobin (HGB) elevation before and after treatment. Simultaneously, the clinical effects of iron supplementation with iron sucrose were compared to those of inpatient patients during the same period.

**Results:**

Baseline values were comparable in both groups. Within 12 weeks of treatment, the elevated HGB level in the ferric derisomaltose group was higher than that of the iron sucrose group, with a statistical difference at all time points, and the proportion of HGB increased over 20 g/L in the patients treated for 4 weeks was higher (98.7％, 75.9％). During the treatment with ferric derisomaltose and iron sucrose, the proportion of mild adverse reactions in the ferric derisomaltose group was slightly lower than that of the iron sucrose group, and neither group experienced any serious adverse reactions. The patients responded well to the infusion treatment, with no reports of pain or pigmentation at the injection site.

**Conclusion:**

The treatment of IDA patients with ferric derisomaltose has a satisfactory curative effect, with the advantages of rapidity, accuracy, and safety. Therefore, it is worthy of widespread clinical use.

贫血导致患者体内组织器官的正常供氧受到影响和阻碍，引发乏力、头晕等多种症状，严重时可导致心力衰竭及死亡。缺铁性贫血（Iron deficiency anemia, IDA）是最常见的贫血类型，约占贫血的60％[1]。近年研究显示，全球IDA病例超过了12亿，给社会带来了巨大的负担[2]。

目前IDA的治疗主要有口服铁剂和静脉铁剂。口服铁剂服用方便，但存在胃肠道不良反应、吸收障碍、纠正贫血速度慢、依从性差等缺陷[3]；而静脉铁剂可更快补充铁元素，能够更快地升高HGB水平，改善贫血症状，已在多种疾病贫血并发症治疗中展现出了治疗优势，大量的研究支持静脉铁剂的疗效与安全性[4]–[6]。

既往常规静脉铁剂单次剂量为100～200 mg，每周不超过3次给药，对于铁需求量≥1 000 mg的患者，需要10 d以上输注，患者依从性差。异麦芽糖酐铁单次最高输注剂量达20 mg/kg，可一次纠正铁缺乏。我们回顾性总结了近2年单中心120例IDA患者应用异麦芽糖酐铁的临床数据，评价疗效及安全性如下。

## 病例与方法

1. 病例：回顾性分析我院2021年6月至2023年3月期间应用静脉异麦芽糖酐铁的IDA患者，诊断标准参考《血液病诊断及疗效标准（第4版）》[7]。同时选取同期我院住院接受蔗糖铁治疗的、年龄及性别匹配的IDA患者作为对照。入组患者中，造成IDA的原因主要为饮食限制导致营养不良，常饮茶、咖啡或胃肠道疾病导致铁吸收障碍，或育龄期女性月经量大导致的失血过多等。通过改变饮食习惯或就诊相应科室进行去因治疗，均已去除病因。

2. 治疗方法：异麦芽糖酐铁固定给药剂量1 000 mg，无需过敏测试，应用250 ml的0.9％无菌氯化钠注射液稀释后单次滴注；蔗糖铁100 mg/d单次静脉输注，连用多次。

3. 随访及疗效评估：通过电话和门诊进行随访，随访截止日期为2023年3月20日。主要评估患者治疗前后HGB水平、治疗4周HGB升高20 g/L患者比例，平均红细胞体积（MCV）、平均红细胞血红蛋白含量（MCH）、平均红细胞血红蛋白浓度（MCHC）变化。定义治疗后男性HGB≥120 g/L，女性HGB≥110 g/L为HGB达标。同时评估患者输注异麦芽糖酐铁过程中及之后有无不良反应，治疗过程中患者不良反应发生率。

4. 统计学处理：采用SAS 9.4软件进行统计分析。患者一般特征分析，计数资料采用*χ*^2^检验，计量资料如服从正态分布采用均数±标准差表示，且满足方差齐性的条件采用*t*检验或单因素方差分析，否则采用中位数（*IQR*）表示，采用非参数检验。连续型结局的组间比较采用重复测量的线性混合模型，固定效应包括性别、年龄、时间和组别×时间交互作用，患者ID为随机效应。各结局随访数据的缺失值采用基于单调回归的多重插补法，考虑的协变量包括性别、年龄、时间、组别×时间交互作用和对应结局的基线值。分类结局（第4周HGB达标率）采用卡方检验进行组间比较。双侧*P*<0.05为差异有统计学意义。

## 结果

一、患者基本资料

2021年6月至2023年3月我院共120例IDA患者应用异麦芽糖酐铁进行治疗，考虑输血对观察铁剂治疗效果有一定影响，故去除住院后有输血史的患者，并排除无随访结果的患者，最终纳入患者86例。同期我院年龄及性别匹配的应用蔗糖铁治疗的IDA患者30例。两组患者基线临床特征见表1。异麦芽糖酐铁组均给药1次，输注量为1 000 mg；蔗糖铁组中位给药次数为10（7～14）次，中位输注量为1 000（700～1 400）mg。

**表1:** 接受异麦芽糖酐铁及蔗糖铁治疗的缺铁性贫血患者基线临床特征比较

基线临床特征	异麦芽糖酐铁组（86例）	蔗糖铁组（30例）	统计量	*P*值
年龄[岁，*M*（范围）]	41（26~84）	38（17~66）	1.385	0.166
性别（例，男/女）	17/69	3/27	1.487	0.223
HGB[g/L，*M*（范围）]	76（46~108）	78（66~95）	0.852	0.394
MCV[fl，*M*（范围）]	71.5（56.2~88.1）	71.2（66.2~80.6）	0.265	0.791
MCH[pg，*M*（范围）]	19.7（15.5~29.6）	21.5（18.9~27.5）	3.705	<0.001
MCHC[g/L，*M*（范围）]	274（243~311）	303（289~315）	6.745	<0.001
SF[ng/ml，*M*（范围）]	3.5（1.0~50.9）	5.5（2.6~10.5）	1.408	0.159
血清铁[Iron，*M*（范围）]	2.8（1.2~5.9）	4.3（1.7~8.4）	3.423	0.001
TIBC[mmol/L，*M*（范围）]	89.1（53~120.3）	94.2（82.2~106.8）	2.450	0.014
TSAT[%，*M*（范围）]	3.1（1.6~7.6）	4.7（1.6~8.5）	8.134	<0.001

注 MCV：平均红细胞体积；MCH：平均血红蛋白含量；MCHC：平均血红蛋白浓度；SF：血清铁蛋白；TIBC：总铁结合力；TSAT：转铁蛋白饱和度

二、疗效

异麦芽糖酐铁组第2、4、8、12周的HGB水平与基线相比显著增高，蔗糖铁组第2、4、8、12周的HGB水平与基线相比也显著增高，第2、4、8、12周各个时间点异麦芽糖酐铁组的HGB相对基线增幅均显著高于蔗糖铁（均*P*<0.001）（表2）。

MCV、MCH和MCHC等贫血参数在12周内的变化结果显示，异麦芽糖酐铁组和蔗糖铁组MCV、MCH和MCHC逐渐增加至正常范围，但是异麦芽糖酐铁组MCV、MCH和MCHC治疗后的水平显著高于蔗糖铁组（均*P*<0.001）（表2）。

**表2:** 接受异麦芽糖酐铁及蔗糖铁治疗的缺铁性贫血患者2、4、8、12周HGB、MCV、MCH、MCHC变化

指标	异麦芽糖酐铁组	*P*值^a^	蔗糖铁组	*P*值^a^	组间差值	*P*值^b^
HGB[g/L, *M*（*IQR*）]						
基线	77.3（74.6, 80.0）		79.2（74.7, 83.6）		−1.9（−6.8, 3.1）	0.462
2周	101.1（98.7, 103.5）	<0.001	90.7（86.7, 94.6）	<0.001	10.4（6.1, 14.8）	<0.001
4周	116.4（113.9, 118.9）	<0.001	107.6（103.6, 111.7）	<0.001	8.8（4.2, 13.3）	<0.001
8周	132.0（130.0, 134.0）	<0.001	121.0（117.8, 124.2）	<0.001	11.0（7.6, 14.4）	<0.001
12周	139.7（137.7, 141.7）	<0.001	131.5（128.3, 134.6）	<0.001	8.2（4.8, 11.6）	<0.001
MCV[fl, *M*（*IQR*）]						
基线	71.5（70.3, 72.7）		72.1（70.1, 74.1）		−0.6（−2.8, 1.7）	0.628
2周	79.8（78.6, 80.9）	<0.001	73.3（71.5, 75.2）	0.040	6.4（4.4, 8.5）	<0.001
4周	83.4（82.3, 84.5）	<0.001	75.0（73.3, 76.7）	<0.001	8.4（6.5, 10.3）	<0.001
8周	86.9（85.9, 87.9）	<0.001	79.8（78.2, 81.4）	<0.001	7.1（5.4, 8.8）	<0.001
12周	89.8（88.9, 90.7）	<0.001	85.0（83.5, 86.4）	<0.001	4.8（3.2, 6.4）	<0.001
MCH[pg, *M*（*IQR*）]						
基线	19.9（19.3, 20.6）		22.0（21.0, 23.1）		−2.1（−3.3, −0.9）	<0.001
2周	23.4（22.9, 24.0）	<0.001	23.0（22.1, 24.0）	<0.001	0.4（−0.6, 1.5）	0.428
4周	25.8（25.3, 26.3）	<0.001	24.7（23.8, 25.5）	<0.001	1.1（0.2, 2.1）	0.014
8周	27.8（27.4, 28.2）	<0.001	27.2（26.6, 27.8）	<0.001	0.6（−0.1, 1.2）	0.116
12周	29.5（29.1, 29.9）	<0.001	28.9（28.2, 29.6）	<0.001	0.6（−0.2, 1.3）	0.117
MCHC[g/L, *M*（*IQR*）]						
基线	276.5（273.5, 279.5）		300.3（295.3, 305.3）		−23.8（−29.4, −18.2）	<0.001
2周	292.7（289.9, 295.5）	<0.001	303.6（299.0, 308.2）	0.046	−10.9（−16.1, −5.7）	<0.001
4周	309.5（307.4, 311.6）	<0.001	307.7（304.3, 311.1）	0.001	1.8（−1.9, 5.5）	0.351
8周	318.7（316.9, 320.5）	<0.001	323.2（320.3, 326.1）	<0.001	−4.4（−7.5, −1.4）	0.005
12周	328.5（326.5, 330.6）	<0.001	325.5（322.2, 328.8）	<0.001	3.1（−0.5, 6.6）	0.096

注 MCV：平均红细胞体积；MCH：平均血红蛋白含量；MCHC：平均血红蛋白浓度。^a^*P*值为与基线水平比较，^b^*P*值为两组间比较

给药4周后，异麦芽糖酐铁组与蔗糖铁组比较，HGB升高≥20 g/L患者比例分别为98.7％、75.9％；HGB达标率分别为62.0％、31.0％，差异有统计学意义（*χ*^2^＝8.194，*P*＝0.004）。

三、安全性分析

使用异麦芽糖酐铁的120例患者中，5例（4.2％）在输注过程中出现轻度不良反应，其中2例出现轻微的颜面部皮肤潮红症状，停止输注后15 min症状消失；2例出现轻度皮疹，对症治疗后好转；1例输注出现胸闷憋气，停止输注并吸氧后症状得以缓解。上述患者均在反应消失后，经过患者同意，再次尝试输注异麦芽糖酐铁，再次开始时降低滴注速度，确保患者耐受良好后的10～15 min，加快输注速度并顺利完成输注。使用蔗糖铁的30例患者中，2例（6.7％）在输注过程中出现轻度不良反应，其中1例出现轻度皮疹，对症后好转；1例出现胸闷憋气，停止输注并吸氧后症状缓解。

## 讨论

IDA是可发生于各年龄段且最为常见的贫血。常见的IDA高危人群包括儿童、经期女性、孕妇、各类器质性疾病患者。2016年全球疾病负担研究中显示：IDA全球患病率列第5位，全球共有12亿人口罹患IDA。IDA可能对多个系统产生影响，包括对心血管疾病的影响，增加围手术期死亡风险、早产、孕妇抑郁风险等[8]–[10]，而且还是导致伤残损失寿命年最高的疾病之一[2]。尽早纠正IDA，对患者预后、疾病管理过程至关重要，当出现IDA时应当及时纠正。目前静脉铁剂可更快补充铁元素、升高HGB水平、改善贫血症状，尤其针对口服铁剂不耐受或依存性差的患者，可迅速满足患者需求[11]–[12]。

《静脉铁剂应用中国专家共识（2019年版）》[6]建议加强静脉铁剂的更广泛应用，尤其对于一些特殊患者和特殊情况下不适用于口服铁剂的患者更推荐使用静脉铁剂。异麦芽糖酐铁注射液是新一代高剂量注射铁剂，免疫原性低，单次可进行足剂量补铁，提高便利性和患者依从性，避免反复注射中可能产生的风险[13]。异麦芽糖酐铁是英国血液病学学会指南唯一批准使用简化表的注射用铁剂[14]。

本研究中，两组患者基线HGB水平偏低，均低于80 g/L。国外研究中，使用静脉铁剂患者基线HGB普遍更高，这也是由于国内外对贫血严重程度分级存在差异，WHO定义HGB<110 g/L属于中度贫血，当HGB<80 g/L属于重度贫血。而中国中重度贫血的阈值更低，HGB<90 g/L属于中度贫血，HGB<60 g/L属于重度贫血[15]–[16]。中重度贫血可导致全身各脏器缺血缺氧表现，长时间中重度贫血会造成重要器官的血流灌注量的不足，容易发生脑血栓、心力衰竭、急性肾功能衰竭、认知功能障碍等疾病，严重者甚至出现低血容量性休克、呼吸循环衰竭及死亡。对于中重度贫血的患者更适合使用静脉铁剂快速提升HGB，最大限度减少贫血对身体的危害。但研究调查显示，国内即使极重度贫血患者得到治疗者不超过一半[17]–[20]。因此，我们需要重视国内贫血的诊断与治疗。

研究结果发现，相比蔗糖铁，异麦芽糖酐铁组改善HGB速度更快，幅度更高，在各个时间点均优于蔗糖铁。HGB应答分析中，提升超过20 g/L作为标准，参考异麦芽糖酐铁相关的PROVIDE研究[21]及其他铁剂的研究[22]–[23]。我们同样做了此分析，结果显示异麦芽糖酐铁治疗4周后HGB提升超过20 g/L的比例为98.7％，显著优于蔗糖铁的75.9％。PROVIDE研究中，患者基线HGB平均值为94 g/L，而本研究中患者基线HGB为76 g/L，因此，本研究中结果显示HGB在4周内提升速度更快，幅度更高，这也提示异麦芽糖酐铁使中度至重度IDA患者的受益更为显著。

另一方面，由于IDA患者对贫血的重视度不足，导致治疗随访不足。面对IDA未得到充分纠正的结果，作为医师应鼓励患者积极规律随访。铁剂选择中，目前临床上仍以口服铁剂为主，但是从本研究中可以看到，大部分IDA就诊患者为女性，其中，月经量大造成的频繁失血是导致IDA主要原因，而口服铁剂对于失血量超过补充速度的患者治疗效果欠佳。《铁缺乏症和缺铁性贫血诊治和预防的多学科专家共识（2022版）》 [5]指出，新一代的静脉注射铁制剂中，铁与碳水化合物结合紧密，实现铁的控制释放，可以在短时间内给予大剂量铁剂。新一代的静脉铁制剂已经改变了IDA的治疗。因此，如何选择合适的IDA治疗方案，需根据患者具体情况并结合指南及共识推荐进行选择。

便捷性及患者依从性来看，异麦芽糖酐铁明显优于二代静脉铁剂蔗糖铁，异麦芽糖酐铁注射液只需要1次输注即可满足大部分患者的治疗剂量。本中心研究中二者平均给药剂量基本一致，同等剂量输注情况下，异麦芽糖酐铁可实现单次输注，这对于无法多次往返医院输液的患者而言非常便利，也有助于节省更多输液门诊空间和时间。而输注1 000 mg蔗糖铁至少需要5～10次，由于门诊输液的限制与住院时长的限制，实现1 000 mg或以上剂量输注的患者比例很小。从全国层面来看，大型真实世界研究显示[24]，蔗糖铁在临床的平均使用剂量仅为510.6 mg，存在补铁剂量不足。

安全性上，Pollock等[25]发表较新的一项大型静脉铁剂安全性Meta分析，统计了21个前瞻性临床研究，涉及8 599例患者的安全性数据，结果显示异麦芽糖酐铁在严重输液反应方面与蔗糖铁相比，异麦芽糖酐铁引起严重过敏反应的风险低49％。另一项最新Meta分析，纳入10 467例患者的数据显示，异麦芽糖酐铁的严重不良反应发生率仅为0.14％，相比于另一种第三代静脉铁剂羧基麦芽糖铁，严重不良反应发生率降低87％[26]。我中心数据显示静脉异麦芽糖酐铁治疗IDA过程顺利，患者耐受性良好，无严重不良反应发生，仅有1例患者出现憋气，暂停输注后对症治疗，待症状缓解降低速度仍完成了治疗。其他轻度输液反应的症状在停止输注后可自行消退，并最终都顺利完成输注。在目前静脉铁剂的输液管理中，我中心遵循国际公认的静脉铁剂不良反应即时管理流程[27]。对于一些开始输注静脉铁剂表现出的过敏症状，但停药后自行消退，同时再次给药不会再次出现的症状，称为补体激活相关的假性过敏反应（complement activation-related pseudo-allergy, CARPA），它与IgE介导的过敏反应最大的差别是反复接触时症状不会再次出现[28]。因此，当出现轻度输液反应时，要正确识别不良反应表现类型，当症状缓解后，可再次尝试。

本研究存在以下局限性：第一，为回顾性研究，并非严格随机对照试验，研究质量具有一定局限，但结果对临床有一定参考意义。后续希望可以进行更高质量的前瞻性研究设计。第二，按照专家共识中使用Ganzoni公式或者异麦芽糖酐铁的说明书中对铁需求量计算，大多数IDA患者对铁需求量大约为1 500 mg[5]–[6]，本研究对患者的静脉补铁量均约为1 000 mg，这可能对疗效有一定影响。同时，本研究只随访观察了12周内的补铁效果，并没有更长期的跟踪随访。有研究已经证明，一次足量补充异麦芽糖酐铁，对于1年甚至更长期的随访结果来看，相比未足量补铁患者，IDA再就诊率大幅降低[29]–[30]。

综上所述，通过静脉滴注异麦芽糖酐铁治疗IDA疗效满意，具有见效迅速、疗效确切、安全性高的优点，同时由于治疗频率低、周期短，大大降低了医疗成本，并且改善了临床上对IDA的管理，值得临床广泛应用。
